# Construction and Investigation of an LINC00284-Associated Regulatory Network in Serous Ovarian Carcinoma

**DOI:** 10.1155/2020/9696285

**Published:** 2020-01-21

**Authors:** Shasha Wang, Lu Zhang, Lin Tao, Lijuan Pang, Ruiting Fu, Yu Fu, Weihua Liang, Yusong Ding, Wei Jia

**Affiliations:** ^1^Department of Pathology, The First Affiliated Hospital, Shihezi University School of Medicine/Department of Pathology, Shihezi University School of Medicine/Key Laboratory for Xinjiang Endemic and Ethnic Diseases, Shihezi, China; ^2^Department of Obstetrics and Gynecology, The First Affiliated Hospital School of Medicine, Shihezi University, Xinjiang 832002, China; ^3^Department of Preventive Medicine, School of Medicine, Shihezi University, Xinjiang 832002, China

## Abstract

The low survival rate associated with serous ovarian carcinoma (SOC) is largely due to the lack of relevant molecular markers for early detection and therapy. Increasing experimental evidence has demonstrated that long noncoding RNAs (lncRNAs) are involved in cancer initiation and development, and a competitive endogenous RNA (ceRNA) hypothesis has been formulated. Therefore, the characterization of new lncRNA and lncRNA-related networks is crucial for early diagnosis and targeted therapy of SOC. Data on lncRNAs, mRNAs, and miRNAs with differential expression in SOC, compared to normal ovarian tissue, were obtained from the Gene Expression Omnibus (GEO) database. Data on lncRNA expression and clinical data in SOC were obtained from The Cancer Genome Atlas (TCGA). lncRNA-miRNA interactions were predicted by the miRBase database. Different online tools, i.e., TargetScan, RNA22, miRmap, microT, miRanda, StarBase, and PicTar, were cooperatively utilized to predict the mRNAs targeted by miRNAs. The plugin of BiNGO in Cytoscape and KOBAS 3.0 were used to conduct the functional and pathway enrichment analyses. The lncRNA, miRNAs, and mRNAs identified to be expressed at statistically significant and different levels between SOC and healthy fallopian tube tissues were further validated using qRT-PCR. A total of 4 lncRNAs (LINC00284, HAGLR, HCAT158, and BLACAT1) and 111 mRNAs were found to be upregulated in SOC tissues compared to normal tissues, based on the GEO database. LINC00284 was found to be highly expressed in SOC, in association with the upregulation of the transcription factor SOX9. The high LINC00284 expression was associated with poor prognosis and proved to be an independent risk factor in patients with SOC, based on TCGA database. The qRT-PCR validation results closely recapitulated the expression profiles and prognostic scores of the aforementioned bioinformatic analyses. The LINC00284-related ceRNA network was found to be associated with SOC carcinogenesis by biofunctional analysis. In conclusion, the LINC00284-related ceRNA network may provide valuable information on the mechanisms of SOC initiation and progression. Importantly, LINC00284 proved to be a new potential prognostic biomarker for SOC.

## 1. Introduction

Ovarian carcinoma (OC) is one of the most common malignancies of the female genital organs, the eighth most lethal female cancer worldwide, and the most lethal gynecological malignancy in developed countries [1]. Serous ovarian carcinoma (SOC) is the most common subtype, accounting for 75-80% of epithelial ovarian carcinomas (EOCs). Due to the lack of effective biomarkers for early detection, approximately 75% of SOC patients present with advanced-stage disease at diagnosis, which results in poor prognosis [2]. Thus, exploring novel biomarkers of SOC progression and prognosis, as well as alternative therapeutic targets, is crucial to improving patient management.

Long noncoding RNAs (lncRNAs) are a class of noncoding transcripts greater than 200 nt in length, which are involved in many biological processes such as chromatin recombination, transcriptional gene expression, and posttranscriptional regulation. lncRNAs play various roles in the regulation of gene expression, serving as “signals,” “decoys,” “guides,” and “scaffolds” [3, 4]. There is accumulating evidence that lncRNAs are involved in the initiation and development of many types of carcinoma, including EOC. For instance, lncRNA TPT1-AS1 [5, 6], lncRNA TPT1-AS1 [7, 8], and HOXD-AS1 [9] were reported to be upregulated in EOC and to promote EOC proliferation and migration. The most common mechanism by which lncRNAs are believed to regulate the expression of target genes involves their role as ceRNAs [10].

In the last decade, signaling networks formed by lncRNA and miRNA molecules were found to coordinate the regulation of gene expression. According to the ceRNA hypothesis, mammalian lncRNAs function as “miRNA sponges,” which competitively bind to miRNAs to antagonize them. This represents one of the “decoy” mechanisms [11]. The ceRNA hypothesis suggests that a variety of RNA molecules form interaction networks, in which lncRNAs, miRNAs, and mRNAs are in a dynamic equilibrium. Alterations in the level of one or more of these molecules affect the expression of the target gene(s), which could lead to tumorigenesis [11].

Here, we comprehensively investigated lncRNA, miRNA, and mRNA sequencing data of SOC and control samples from the Gene Expression Omnibus (GEO) data matrix, to identify aberrantly expressed species. Next, the prognostic value of overexpressed lncRNAs (LINC00284, HAGLR, HCAT158, and BLACAT1) was assessed in patients with SOC, based on TCGA database. Finally, transcription factors (TFs) positively associated with LINC00284 expression were identified.

## 2. Materials and Methods

### 2.1. Data Collection

Gene Expression Omnibus (GEO) datasets including GSE18520, GSE36668, GSE119055, and GSE83693 were downloaded from the GEO database (https://www.ncbi.nlm.nih.gov/geo/). Specifically, 61 serous ovarian carcinomas and 14 normal ovarian surface epithelium tissues were used for lncRNA and mRNA data analysis in the GSE18520 and GSE36668 datasets; 22 serous ovarian carcinomas and 7 normal ovarian surface epithelium tissues were used for miRNA data analysis in the GSE119055 and GSE83693 datasets.

### 2.2. Analysis of Differentially Expressed Genes

Differential expression analysis was carried out to identify differentially expressed lncRNAs, mRNAs, and miRNAs between SOC and normal tissues by using the R/Bioconductor package of edgeR, setting a cutoff value of ∣log2FC∣ > 2 (FC, fold change) and a *P* value < 0.01 as the statistical significance threshold.

### 2.3. Survival Analysis Based on TCGA Data

For survival analysis in TCGA SOC patients, high-throughput sequencing LINC00284 expression data (ending date: January 28, 2016) from 371 SOC samples were downloaded using R software (R 3.4.2). The “RTCGAToolbox” library was used for this analysis. The best cutoff value of LINC00284 RNA expression was used as the cutoff value to divide the samples into high- and low-expression groups. The median, minimum, and maximum LINC00284 expression values were 1.11, 0, and 29.65, respectively. The publication guidelines of TCGA Research Network were followed in this study (https://cancergenome.nih.gov/publications/publicationguidelines). Thus, no further ethical approvals were required.

### 2.4. Kaplan-Meier Plotter Online Platform

TCGA and GEO SOC datasets were selected using the Kaplan-Meier plotter online platform (http://kmplot.com/analysis/). LINC00284 RNA expression was determined using the 232318_s_at probe (the same probe was used for the GEO database, so that the datasets were comparable). The best cutoff value for LINC00284 RNA expression was automatically selected by the online platform. A total of 614 SOC samples were analyzed for progression-free survival, whereas 356 and 380 samples from SOC patients treated with a combination of taxol and platin were employed for overall survival and progression-free survival analyses, respectively.

### 2.5. lncRNA-miRNA-mRNA Network Construction

lncRNA-miRNA interactions were predicted via the miRBase database (http://www.mirbase.org). TargetScan (http://www.targetscan.org/), RNA22 (https://cm.jefferson.edu/rna22/Interactive/), miRmap (http://mirnamap.mbc.nctu.edu.tw/), microT (http://diana.imis.athena-innovation.gr/DianaTools/index.php?r=microT_CDS/index), miRanda (http://mirdb.org/index.html), StarBase (http://starbase.sysu.edu.cn/), and PicTar (https://pictar.mdc-berlin.de) databases were cooperatively utilized to predict the mRNA targets of the miRNAs. Cytoscape (version 3.5.1) was utilized to build and visualize the miRNA-mRNA network based on the identified lncRNA/miRNA and miRNA/mRNA interactions [12].

### 2.6. Functional Annotation

The BiNGO plugin in Cytoscape (version 3.5.1) and KOBAS 3.0 (http://kobas.cbi.pku.edu.cn/) were used to conduct the functional and pathway enrichment analyses. Gene Ontology (GO) and Kyoto Encyclopedia of Genes and Genomes (KEGG) pathway enrichment analyses were performed to assess the potential biological functions and pathways of the overexpressed mRNAs included in the network (*P* value <0.05).

### 2.7. Preparation of Human SOC Samples

In total, 40 and 20 formalin-fixed, paraffin-embedded SOC and healthy fallopian tube tissue specimens (one from each patient), respectively, were obtained from the Department of Pathology of the First Affiliated Hospital of Shihezi University School of Medicine. The collection of specimens was approved and supervised by the Ethics Committee of the First Affiliated Hospital of Shihezi University School of Medicine. Clinical data of patients with SOC, including age, recurrence-free survival, and overall survival, were collected from the on-paper medical records at the First Affiliated Hospital of Shihezi University and from the electronic medical record system. Recurrence-free survival was defined as the time from surgery to relapse or until the study endpoint. Overall survival was calculated as the time from surgery to death or until the endpoint of the study, that is, March 5, 2019. No patients in this study received chemotherapy or radiotherapy before surgery. The optimal cutoff value (4.791) of LINC00284 RNA expression was used to sort the samples into high- and low-expression groups. The median, minimum, and maximum LINC00284 expression values were 6.566, 1.516, and 101.125, respectively.

### 2.8. RNA Extraction and qRT-PCR

Total RNA was extracted from tissues using the TRIzol reagent and the miRNeasy FFPE Kit (Qiagen, Valencia, CA). The QuantiTect Reverse Transcription Kit (Qiagen) and a QuantiFast SYBR Green PCR kit (Qiagen) were used to synthesize cDNA and perform quantitative real-time polymerase chain reaction (qRT-PCR) analysis on a 7500 Fast Real-Time PCR System (Life Technologies, Shanghai, China). The results of LINC00284, SOX9, MYB, ESRP1, and hsa-miR-195-5p and hsa-miR-497-5p were normalized to the expression of GAPDH and U6, respectively. The primer sequences were as follows: LINC00284—forward primer (5′-3′): GCAAACCACCTCACCACACTATCC and reverse primer (5′-3′): CCAAGTCACGCTGTCATGCCTAG; miR-195-5p—forward primer (5′-3′): AGCTTCCCTGGCTCTAGCAG and reverse primer (5′-3′): ATTGGCAGACTCGCTTCCCT; miR-497-5p—forward primer (5′-3′): GGTTTGTACGGCACTGTGGC and reverse primer (5′-3′): CCACCCTCGCTCTAACACCA; SOX9—forward primer (5′-3′): CACACGCTGACCACGCTGAG and reverse primer (5′-3′): GCTGCTGCTGCTCGCTGTAG; MYB—forward primer (5′-3′): CCATTGCCGACCACACCAGAC and reverse primer (5′-3′): TTCTTCAGGTAGGGAGCCAGGATC; ESRP1—forward primer (5′-3′): AGCACCGAGACCTAGCACTACAG and reverse primer (5′-3′): TCCTTGGAGAGAAACTGGGCTACC; GAPDH—forward primer (5′-3′): GAGTCAACGGATTTGGTCGT and reverse primer (5′-3′): TTGATTTTGGAGGGATCTCG; and U6—forward primer (5′-3′): CTCGCTTCGGCAGCACA and reverse primer (5′-3′): AACGCTTCACGAATTTGCGT.

### 2.9. Statistical Analysis

A nonparametric test was used to analyze the differences in LINC00284 and SOX9 expression between normal ovarian surface epithelium and SOC tissues. Univariate and multivariate analyses using the Cox regression model were conducted to determine the independent significance of relevant clinical covariates. Survival analysis was performed using the Kaplan-Meier method, and the logrank test was used to analyze the correlation between LINC00284 expression and SOC patient prognosis. All tests were two-sided. *P* < 0.05 was considered significant, and all analyses were performed using the Statistical Product and Service Solutions (SPSS) software (version 20.0; SPSS, Chicago, IL).

## 3. Results

### 3.1. Screening of lncRNAs in GEO Databases

**Figure 1:**
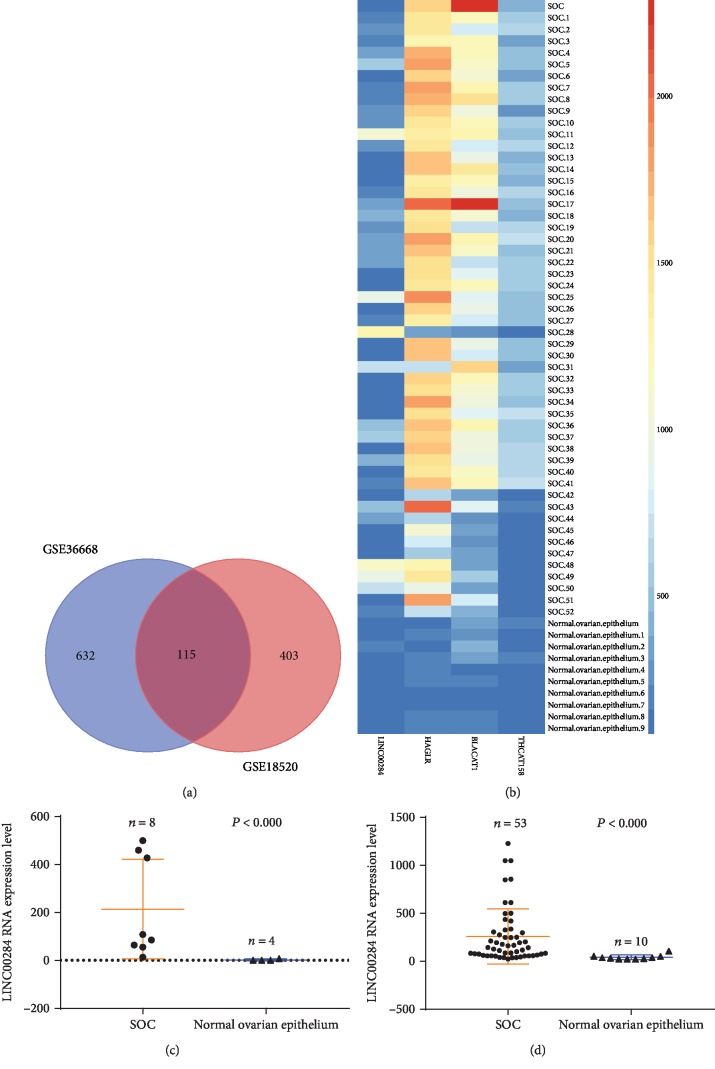
Analysis of gene expression based on 2 GEO datasets (GSE36668 and GSE18520). (a) Venn analysis of lncRNAs and mRNAs upregulated in serous ovarian carcinoma compared to normal tissues from the 2 GEO datasets. (b) Heatmap of the 4 upregulated lncRNAs in 53 SOC tissues compared to 10 normal tissues. Each column represents one sample, and each row indicates one lncRNA. A color scale from blue (low) to red (high) indicates the level of normalized expression. (c, d) Functional plotting of the corresponding mRNA levels based on TCGA database: (c) GSE36668 dataset and (d) GSE18520 dataset. LINC00284 levels were higher in serous ovarian carcinoma samples than in normal samples.

The differential expression of lncRNAs and mRNAs between SOC and normal tissues was separately analyzed in 2 datasets of the GEO database. Genes with a fold change > 2 and *P* value < 0.01 were considered discriminatively expressed. Four lncRNAs were identified in both datasets (LINC00284, HAGLR, HCAT158, and BLACAT1), and 111 mRNAs were found to be upregulated in SOC compared to normal tissues (Figure 1(a)). Expression heatmaps were constructed based on the above lncRNAs (Figure 1(b)). The results suggested that the expression profiles of the upregulated species could distinguish SOC tissues from normal tissues.

### 3.2. Screening of Survival-Related lncRNAs

**Figure 2:**
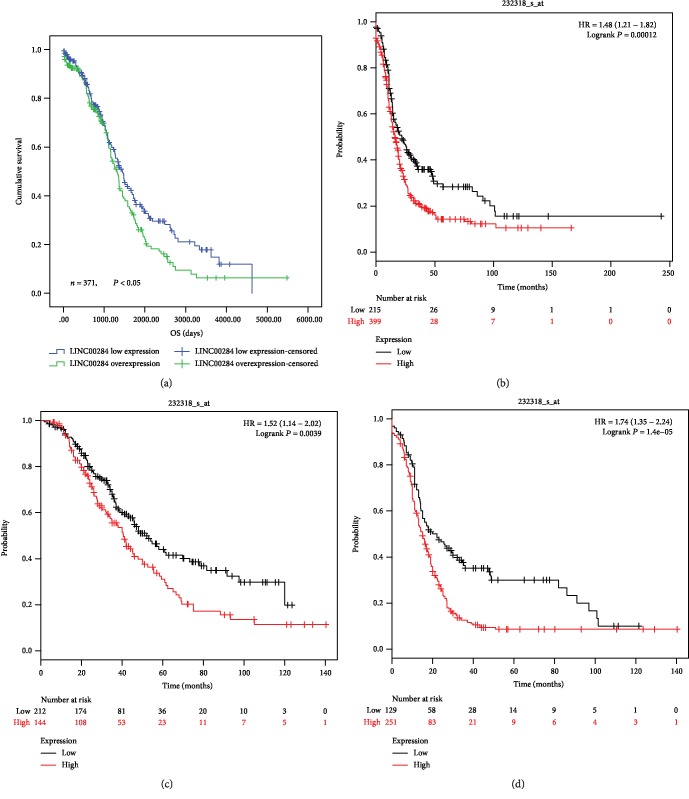
Analysis of the relationship between LINC00284 expression and prognosis in patients with serous ovarian carcinoma based on TCGA (a) and TCGA/GEO (b–d) datasets. (a, b) Patients with LINC00284 overexpression had shorter overall and progression-free survival than those with low LINC00284 expression (*P* < 0.05 and *P* < 0.001, respectively). (c, d) Patients with serous ovarian carcinoma (SOC) and LINC00284 overexpression treated with chemotherapeutic drugs including taxol/platin combinations had shorter overall and progression-free survival (*P* < 0.01 and *P* < 0.0001, respectively).

Survival information on SOC samples from 371 patients was available in TCGA. Receiver operating characteristic (ROC) analysis was used to determine the area under the curve. The (0, 1) point, which maximizes both sensitivity and specificity, could be clearly observed on the ROC curve of each lncRNA expression profile (Supplementary Fig ([Supplementary-material supplementary-material-1])). Therefore, we assigned expression scores of 1.27, 19.75, 0.26, and 4.58 to LINC00284, HAGLR, HCAT158, and BLACAT1, respectively, as optimal cutoffs for survival analyses. The relationship between these 4 lncRNAs and patient prognosis was evaluated by Kaplan-Meier survival analysis (Table 1). The results indicated that LINC00284 overexpression (differential expression of LINC00284; Figures 1(c) and 1(d)) was associated with significantly reduced overall survival (*P* < 0.05; Table 1 and Figure 2(a)). Moreover, based on Kaplan-Meier plotter analysis of TCGA and GEO data, patients with LINC00284 overexpression had shorter progression-free survival than those with low LINC00284 expression (*P* < 0.001, Figure 2(b)). SOC patients with LINC00284 overexpression who were treated with chemotherapeutic drugs that contained taxol and platin together displayed significantly reduced overall and progression-free survival compared to patients with low LINC00284 expression (*P* < 0.01 and *P* < 0.0001, respectively; Figures 2(c) and 2(d)).

### 3.3. LINC00284 Is an Independent Risk Factor for and Prognostic Predictor of SOC

**Figure 3:**
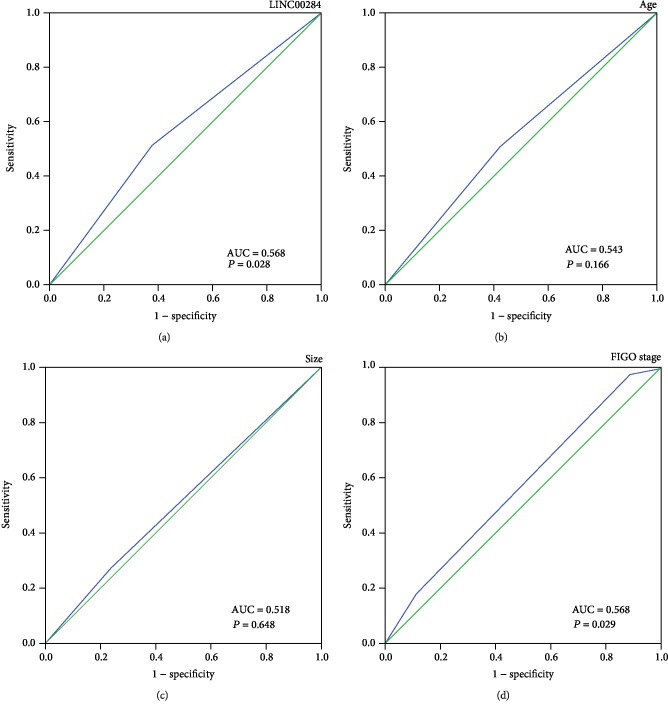
ROC analysis for each clinicopathological parameter was used to determine the cutoff score for the overexpression of LINC00284, based on TCGA dataset. The sensitivity and specificity for each clinicopathological characteristic were plotted: LINC00284 (a) (*P* = 0.028), age (b) (*P* = 0.166), tumor size (c) (*P* = 0.648), and FIGO stage (d) (*P* = 0.029).

Based on univariate analysis using the Cox regression model, LINC00284 overexpression was found to be a strong prognostic factor of poor overall survival (*P* = 0.044; Table 2). In addition, advanced stage (*P* = 0.038) and age (*P* = 0.046) were associated with shorter overall survival. For multivariate Cox regression analysis, only variables that were statistically significant based on univariate Cox regression analysis were considered, and the results identified LINC00284 overexpression (*P* = 0.020), advanced stage (*P* = 0.038), and age (*P* = 0.009) as independent prognostic factors (Table 2). ROC analysis (Figure 3) revealed that the area under the curve of LINC00284 expression (AUC = 0.568, *P* = 0.028; Figure 3(a)) was the same as that of the FIGO stage (AUC = 0.568, *P* = 0.029; Figure 3(d)). Thus, LINC00284 expression exhibited the same prognostic sensitivity and specificity as the FIGO stage.

### 3.4. LINC00284-Related ceRNA Network in SOC

**Figure 4:**
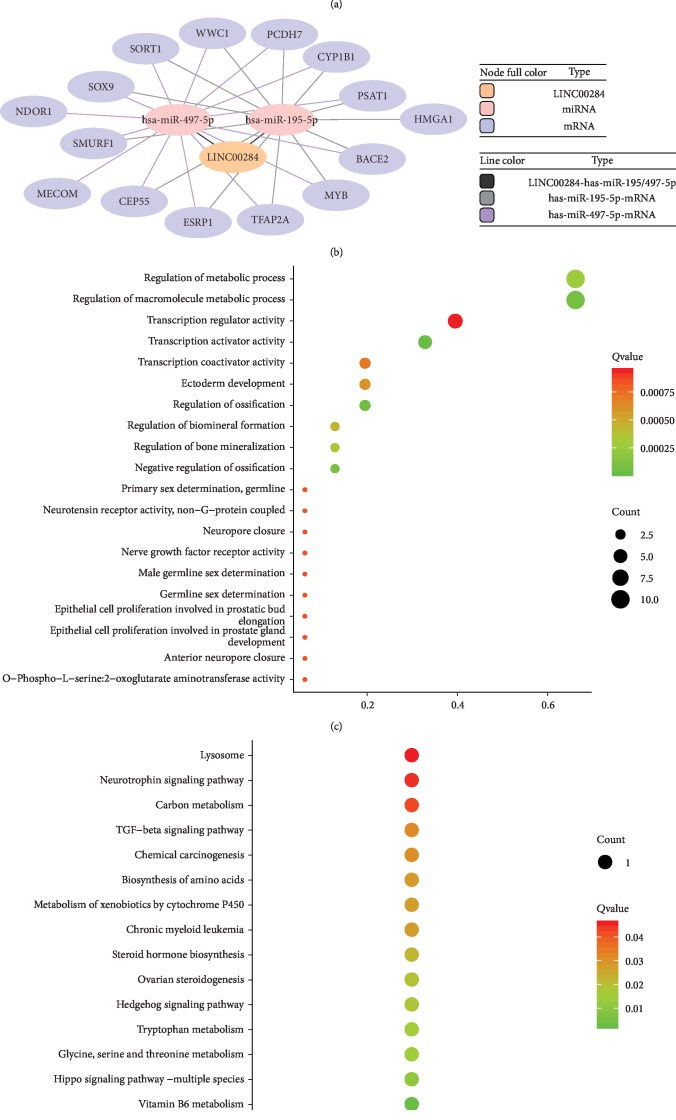
Enrichment analysis of the LINC00284-miRNA-mRNA network. (a) Venn analysis of upregulated miRNAs in SOC compared to normal tissues, based on 2 GEO datasets (GSE83693 and GSE119055). (b) The LINC00284-miRNA-mRNA network in SOC tissues. The node colors represent different RNA types, whereas the line colors represent different LINC00284/miRNA and miRNA/mRNA interactions. (c) Top 20 enrichment Gene Ontology (GO) biological process terms of overexpressed mRNAs involved in the LINC00284-miRNA-mRNA network. The size of balls reflects the gene number, and the color represents the *P* value. (d) Top 15 enrichment KEGG pathways biological process terms of overexpressed mRNAs with *P* value < 0.05 involved in the LINC00284-miRNA-mRNA network. The size of balls reflects the gene number, and the color represents the *P* value.

To explore the function of LINC00284, we screened downregulated miRNAs, based on the ceRNA hypothesis, in 2 GEO datasets (Figure 4(a)). Potential interactions within lncRNA-miRNA-mRNA networks were predicted. Two specific downregulated miRNAs, hsa-miR-195-5p and hsa-miR-497-5p, were predicted to interact with LINC00284 through miRNA response elements, by the miRBase (http://www.mirbase.org/) online tools (Table 3). To improve the predictive accuracy, TargetScan, RNA22, miRmap, microT, miRanda, StarBase, and PicTar databases were combined to identify candidate mRNA targets of the 2 downregulated miRNAs; mRNAs with at least 3 binding sites were selected. As a result, 15 candidate mRNA targets were identified. Finally, a ceRNA network including 1 lncRNA, 2 miRNAs, and 15 mRNAs was visualized, using the Cytoscape software, based on the interactions among LINC00284, miRNAs, and mRNAs indicated in Table 3 (Figure 4(b)).

### 3.5. Functional Analysis of Upregulated mRNAs in the LINC00284-Related ceRNA Network

Functional analysis revealed that the 15 upregulated mRNAs in the above ceRNA network were enriched in 64 GO biological process categories and 15 KEGG categories (*P* < 0.05). The significant GO biological processes of dysregulated genes were regulation of the macromolecule metabolic process (GO: 0060255), regulation of the metabolic process (GO: 0019222), and transcription activator activity (GO: 0016563) (Figure 4(c)).  Figure 4(d) shows the significantly enriched pathways related to these upregulated mRNAs, according to KEGG analysis (Figure 4(d)). Two cancer-related pathways were included, i.e., the TGF-beta signaling and the chemical carcinogenesis pathway.

### 3.6. Screening of LINC00284-Related Transcription Factors (TFs)

**Figure 5:**
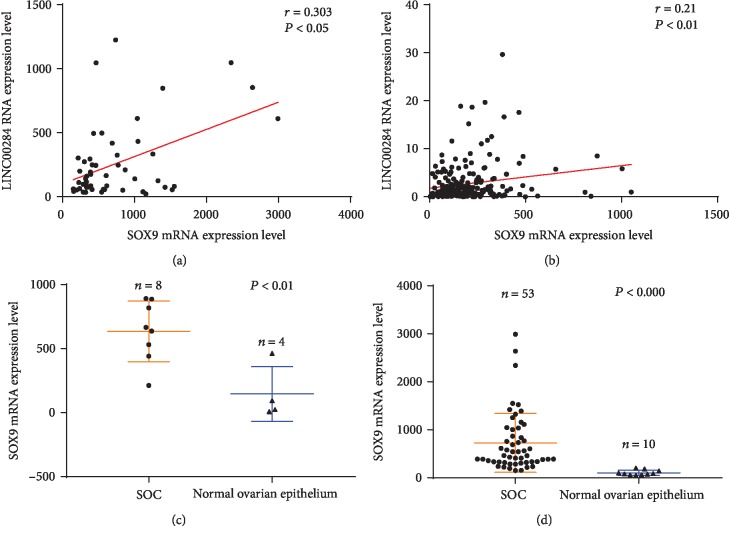
Correlation analysis between SOX9 and LINC00284 expression based on the GEO (a) and TCGA (b) datasets. The expressions of SOX9 and LINC00284 were significantly and positively correlated. Functional plotting of the corresponding mRNA levels based on TCGA database. ((c) GSE36668; (d) GSE18520) SOX9 levels were higher in serous ovarian carcinoma samples than in normal samples.

The transcription of lncRNAs is regulated by specific TFs [13]. Thus, we screened TFs associated with LINC00284 expression. TFs potentially binding to the LINC00284 promoter were identified by JASPAR (http://jaspar.genereg.net/). Using a score > 7 and relative > 0.8 as a screening condition, 357 candidate TFs were identified. Among these, 6 were upregulated in both GEO datasets (SOX9, MYB, TFAP2A, EHF, GRHL2, and ELF3). The correlation of these TFs with LINC00284 was analyzed based on the GEO and TCGA databases (Table 4). The results showed that SOX9 was significantly correlated with LINC00284 in all three datasets (GSE18520 (*P* < 0.05), TCGA (*P* < 0.01); Figures 5(a) and 5(b) and Table 4). The level of SOX9 was significantly higher in SOC compared to normal tissues in the GEO datasets (*P* < 0.01 and *P* < 0.000, respectively; Figures 5(c) and 5(d)).

### 3.7. Confirmation of the Identified Molecules by qRT-PCR

**Figure 6:**
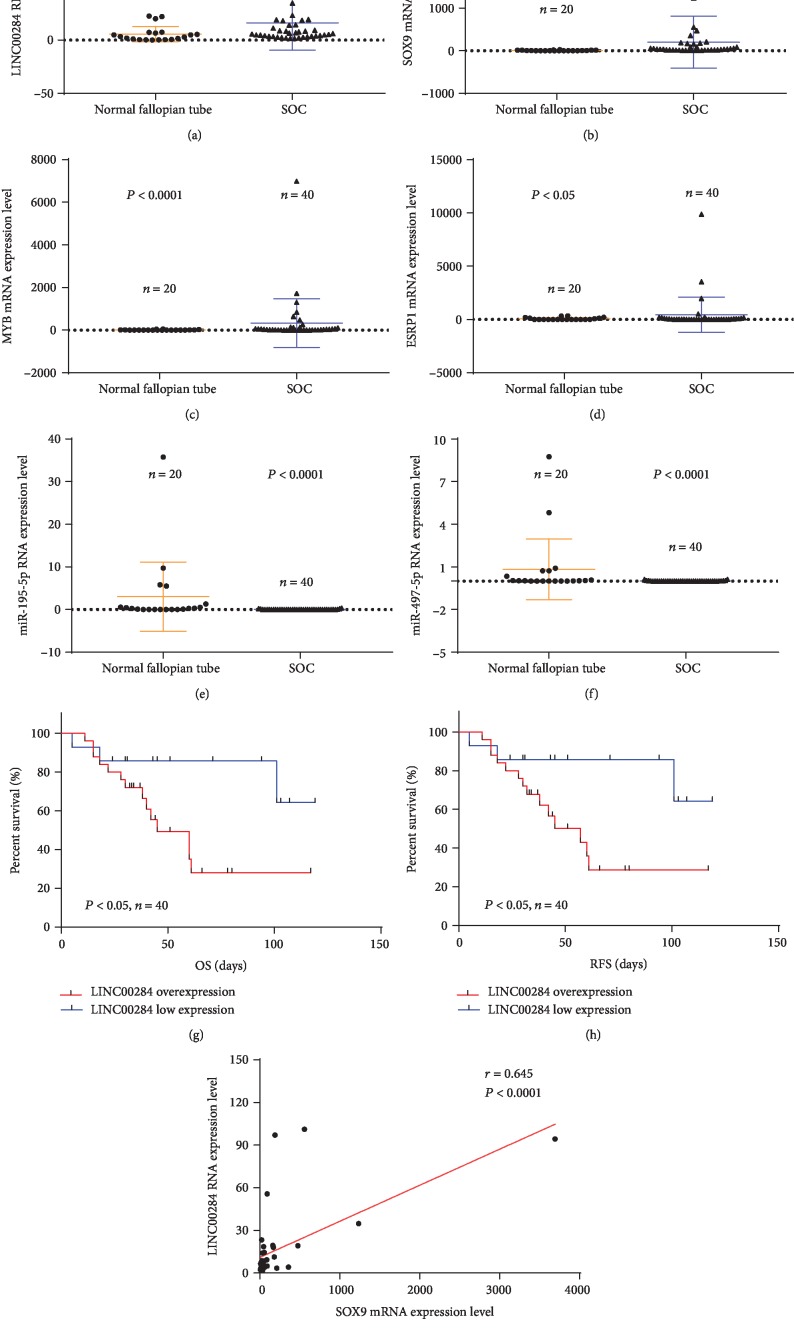
qRT-PCR analysis of the expression of identified molecules. As compared with healthy fallopian tube tissues, (a–d) LINC00284, SOX9, MYB, and ESRP1 are overexpressed in SOC tissues, and (e, f) miR-195-5p and miR-497-5p are expressed at a low level in SOC tissues. (g, h) Kaplan-Meier analysis suggested that patients with LINC00284 overexpression had shorter overall and recurrence-free survival than those with low LINC00284 expression. (i) The expressions of SOX9 and LINC00284 were significantly and positively correlated.

qRT-PCR was used to validate the expressions of identified molecules, including LINC00284, miR-195-5p, miR-497-5p, MYB, ESRP1, and SOX9. Among the 15 upregulated mRNAs capable of binding to miR-195/497-5p, the mRNAs MYB, ESRP1, and SOX9 were selected for further verification because the respective fold changes of these differentially expressed mRNAs were relatively large (FC > 3). Through a comprehensive analysis of the functional analysis results, we inferred that MYB, ESRP1, and SOX9 may play important roles in SOC progression. Our results showed that compared with the normal fallopian tube tissues, LINC00284, SOX9, MYB, and ESRP1 were overexpressed in the SOC tissues (*P* < 0.01, *P* < 0.0001, *P* < 0.0001, and *P* < 0.05, respectively, Figures 6(a)–6(d)) and miR-195-5p and miR-497-5p were expressed at low levels in the SOC tissues (*P* < 0.0001 and *P* < 0.0001, respectively; Figures 6(e) and 6(f)). Meanwhile, Kaplan-Meier analysis suggested that the SOC patients with LINC00284 overexpression showed an expected poorer overall survival and recurrence-free survival than those with low LINC00284 expression (*P* < 0.05 and *P* < 0.05, respectively; Figures 6(g) and 6(h)). In addition, our results also revealed that SOX9 significantly correlated with LINC00284 in SOC tissues (*P* < 0.0001, Figure 6(i)).

## 4. Discussion

Long noncoding regulatory elements, accounting for most of the genome components, are transcribed into lncRNAs located in the nucleus and the cytoplasm. lncRNAs are involved in the regulation of gene expression [4] and affect chromatin modification, X-chromosome silenced genomic imprinting, transcriptional interference and activation, mRNA splicing, mRNA stabilization, and protein translation [14]. Alterations in the expression profile of lncRNAs may be associated with the initiation of specific lesions and may therefore serve as early disease indicators. Indeed, a growing number of lncRNAs were found to be suitable biomarkers for diagnosis and prognosis [15]. Moreover, lncRNAs are also regarded as new potential therapeutic targets.

In the present study, 111 mRNAs and 4 lncRNAs were found to be upregulated in SOC compared to normal tissues, based on the GEO database. Patients with LINC00284 overexpression experienced significantly reduced overall survival compared to patients with low LINC00284 expression, based on TCGA database, which was consistent with the results of Kaplan-Meier plotter analysis of TCGA and GEO data. Based on multivariate analysis using the Cox regression model, LINC00284 overexpression was identified as an independent prognostic factor and was related to SOC development and poor prognosis. In addition, ROC analysis revealed that the area under the curve of LINC00284 expression was the same as that of FIGO staging, demonstrating comparable prognostic sensitivity and specificity. Notably, it has been reported that LINC00284 overexpression in triple-negative breast cancer (TNBC) and cancer stem cells (CSCs) contributes to cancer cell survival and tumor growth [16], which is consistent with our results.

The ability of lncRNAs to regulate mRNA stability and protein translation was also demonstrated [14]. We hypothesized that the mRNAs found to be overexpressed in SOC may be regulated by LINC00284. No protein was predicted to directly bind to LINC00284 by the RPISeq machine learning tool (http://pridb.gdcb.iastate.edu/RPISeq/) and the LncTar software (http://www.cuilab.cn/lnctar). Therefore, we reasoned that LINC00284 could act indirectly on target genes by upregulating the expression of specific mRNAs. miRNAs have been reported to bind to the 3′UTR region of their target genes, thereby decreasing the stability of the target mRNA or downregulating the expression of the related protein [10]. The ceRNA hypothesis postulates that lncRNAs recruit free miRNAs, thereby reducing their abundance and affecting the expression of downstream target genes [10]. We used the GEO database to select mRNAs that were downregulated in SOC, identified potential lncRNA-miRNA-mRNA interaction networks based on the presence of specific binding sites, and reconstructed a comprehensive ceRNA network. Several recent studies demonstrated that ceRNA-based mechanisms may operate in all types of carcinoma [5, 6, 17–23]. In the present study, among the miRNAs found to be downregulated in SOC, hsa-miR-195-5p and hsa-miR-497-5p were predicted to bind to LINC00284. Notably, miRNA-195-5p was also found to be downregulated in human prostate cancer and inhibit cell proliferation and angiogenesis by downregulating PRR11 expression [24]. Moreover, miRNA-497-5p is downregulated in breast cancer, which results in PTEN upregulation and promotion of cell proliferation by competitive binding to HOXC13-AS [7, 8]. Our results predict that both hsa-miR-195-5p and hsa-miR-497-5p could bind to 11 of the 15 mRNAs that were found to be upregulated in SOC. In addition, function analysis revealed that these upregulated mRNAs may relate to tumor occurrence and development, as also previously reported for many cancers, including EOC. For example, SOX9 [25], MYB [26], and ESRP1 [27] promote ovarian cancer cell proliferation. Therefore, we speculated that a dual modulation by miR-497 and miR-195 could underlie SOC pathogenesis. Vidovic and colleagues found that LINC00284 is mainly expressed in the nucleus of breast cancer cells [16].

However, based on our hypothesized ceRNA mechanism, LINC00284 would mainly function in the cytoplasm. The pathogenesis and microenvironment of these two tumors are different, which may account for a different intracellular distribution of LINC00284. Further research is needed to directly verify the intracellular localization of LINC00284 in SOC.

**Figure 7:**
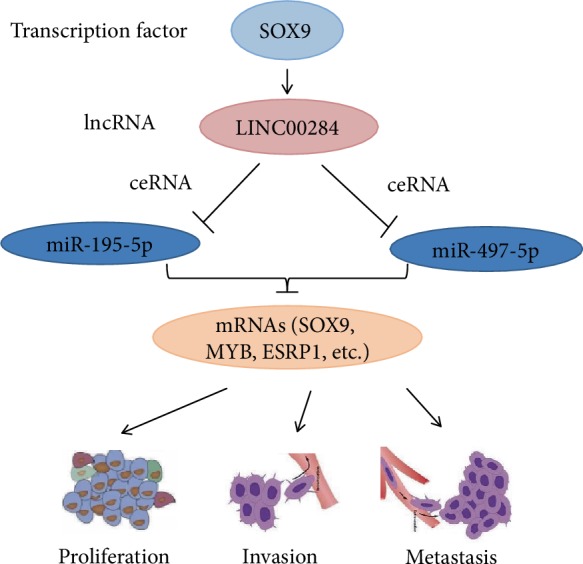
A schematic model of LINC00284 functions in serous ovarian carcinoma. The transcription of LINC00284 is regulated by SOX9. LINC00284 may function as “miRNA sponges,” which competitively bind to miRNA-195/497-5p to antagonize their inhibition of target genes, thereby promoting the expression of target genes (SOX9, ESRP1, ESRP1, etc.) and finally promoting serous ovarian carcinoma initiation and progression.

It was reported that the transcription of lncRNAs is regulated by TFs [13]. We hypothesized that LINC00284 overexpression could be induced by specific TFs. Therefore, we screened TFs that were upregulated in SOC and found that the binding of one of them, SOX9, to the LINC00284 promoter region positively correlated with LINC00284 expression. Of note, in gastric cancer, the upregulation of the transcription factor EGR1 results in enhanced transcription of lncRNA-HNF1A-AS1 and in the promotion of cell proliferation [28]. Based on the present results, we hypothesized that LINC00284 may promote initiation and progression of SOC through the SOX9-LINC00284-miRNA-195/497-5p-mRNA network (Figure 7).

Subsequently, qRT-PCR validation of LINC00284, miR-195-5p, miR-497-5p, MYB, ESRP1, and SOX9 expression and correlation analyses between SOX9 and LINC00284 in 40 SOC tissue samples and 20 healthy fallopian tube tissues were performed. The results of the qRT-PCR validation showed consistent agreement with the expression data available in the GEO and TCGA databases. Next, we analyzed the association between LINC00284 expression and prognosis of the patients with SOC, and the results were similar to the aforementioned bioinformatic analysis results. Therefore, the bioinformatic analysis used in this study can be deemed reliable.

## 5. Conclusions

In conclusion, genome-wide analysis in a cohort of patients with SOC identified various dysregulated lncRNA, miRNA, and mRNA networks from the GEO database. LINC00284 was found to be highly expressed in SOC. LINC00284 upregulation was most likely induced by SOX9 and was associated with poor prognosis, proving to be an independent risk factor in SOC. Therefore, LINC00284 could be a new biomarker for predicting the prognosis of SOC. Further in-depth functional characterization of the LINC00284-related ceRNA network may provide valuable insights into the molecular events responsible for SOC initiation and progression.

## Figures and Tables

**Table 1:** Univariate analysis for overall survival.

Variables	Median OS (months)	*P*
LINC00284 expression		0.045^∗^
Overexpression	90	
Low expression	77	
HAGLR expression		0.827
Overexpression	103	
Low expression	79	
HCAT158 expression		0.015^∗^
Overexpression	72	
Low expression	90	
BLACAT1 expression		0.117
Overexpression	87	
Low expression	130	

Abbreviation: OS: overall survival.

**Table 2:** COX regression analysis of risk factors in patients with serous ovarian carcinoma.

Characteristics	Univariate analysis		Multivariate analysis	
	HR (95% CI)	*P*	HR (95% CI)	*P*
LINC00284 expression				
Low expression	1		1	
Overexpression	1.307 (1.007, 1.696)	0.044^∗^	1.371 (1.051, 1.787)	0.020^∗^
Age				
≤52	1			
>52	1.302 (1.004, 1.688)	0.046^∗^	1.423 (1.092, 1.854)	0.009^∗∗^
Stage				
Early (I-II)	1		1	
Advanced (III-IV)	2.849 (1.059, 7.667)	0.038^∗^	2.848 (1.058, 7.664)	0.038^∗^
Size				
≤20 mm	1			
>20 mm	1.294 (0.954, 1.755)	0.097		

Abbreviations: HR: hazard ratio; CI: confidence interval.

**Table 3:** miRNAs and mRNAs in the LINC00284-related ceRNA network in serous ovarian carcinoma.

lncRNA	miRNAs	mRNAs
LINC00284	hsa-miR-195-5p	SOX9, ESRP1, MYB, WWC1, PSAT1, SORT1, PCDH7, BACE2, CEP55, TFAP2A, CYP1B1, SMURF1, HMGA1
	hsa-miR-497-5p	NDOR1, SOX9, MECOM, ESRP1, MYB, WWC1, PSAT1, SORT1, PCDH7, BACE2, CEP55, TFAP2A, CYP1B1, SMURF1, HMGA1

**Table 4:** Spearman correlation analysis between transcription factors and LINC00284.

TFs	GSE36668		GSE18520		TCGA	
	*r*	*P*	*r*	*P*	*r*	*P*
SOX9	0.643	0.043^∗^	0.303	0.028^∗^	0.21	0.008^∗∗^
MYB	0.524	0.183	-0.009	0.95	-0.067	0.279
TFAP2A	0.19	0.651	-0.162	0.246	-0.086	0.166
EHF	0.619	0.102	0.215	0.122	0.261	0.000^∗∗∗^
GRHL2	0.19	0.651	-0.051	0.716	-0.061	0.325
ELF3	0.095	0.823	0.339	0.013^∗^	0.172	0.006^∗∗^

Abbreviation: TFs: transcription factors.

## Data Availability

All datasets are included in the manuscript.

## Supplementary Materials

Supplementary Materials Supplementary figure: ROC analysis for 4 upregulated lncRNAs was used to determine the cutoff score for the overexpression of lncRNAs, based on TCGA dataset. The sensitivity and specificity for 4 upregulated lncRNAs were plotted: LINC00284 (A) (*P* = 0.037), HAGLR (B) (*P* = 0.330), THCAT158 (C) (*P* = 0.077), and BLACAT1 (D) (*P* = 0.629).
