# Supplementary figures and images for: Construction and Investigation of an LINC00284-Associated Regulatory Network in Serous Ovarian Carcinoma
Source: Dis Markers. 2020 Jan 21;2020:9696285. doi: 10.1155/2020/9696285 (PMC6996679; doi:10.1155/2020/9696285)

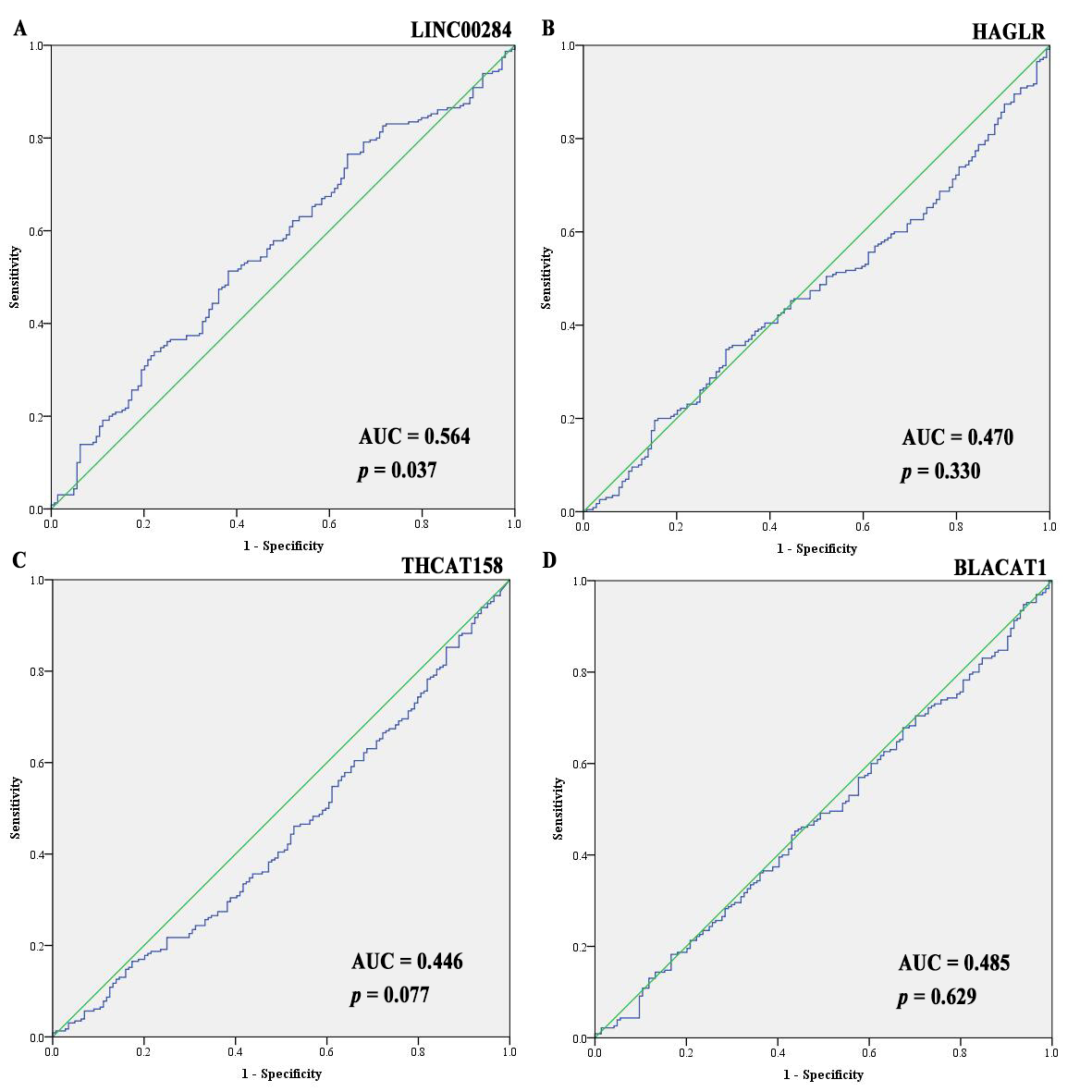

Supplement: Supplementary Materials — Supplementary figure: ROC analysis for 4 upregulated lncRNAs was used to determine the cutoff score for the overexpression of lncRNAs, based on TCGA dataset. The sensitivity and specificity for 4 upregulated lncRNAs were plotted: LINC00284 (A) (P = 0.037), HAGLR (B) (P = 0.330), THCAT158 (C) (P = 0.077), and BLACAT1 (D) (P = 0.629). [file 9696285.f1.tif]
